# Unusual morphology of isolated male epispadia: A rare case report

**DOI:** 10.1016/j.eucr.2024.102707

**Published:** 2024-03-19

**Authors:** Kevin Anthony Glorius Tampubolon, Jupiter Sibarani

**Affiliations:** Department of Urology, Hasan Sadikin Academic Medical Center, Universitas Padjadjaran, Indonesia

**Keywords:** Epispadia, Ambiguous genitalia, Pediatric urology

## Abstract

In less than 10% of cases, males may have isolated epispadias, which is caused by failure in the urethral tubularization process, leading to dorsal urethral defect. This case report presents a unique instance where epispadias was associated with ambiguous genitalia. A 5-year-old boy diagnosed with epispadias. The penis resembled external female genitalia, with scrotal skin covering it. He underwent a two-stage operation without complications. The aim of the surgical techniques is to correct these anomalies and restore urinary continence and sexual function. Long-term outcomes of the surgery can vary, which highlights the need for further research.

## 1 Introduction

Epispadias is a rare congenital anomaly with an incidence rate of 1 per 117,000 males, falling within the bladder exstrophy-epispadias complex (BEEC) spectrum.^1^^,^^2^ Isolated male epispadias constitute <10% of BEEC cases, resulting from the failure of urethral tubularization, leading to a defect in the dorsal part of the urethra.^2^ The urethral meatus in epispadias is large and widely open.^1^^,^^3^ Based on the location, epispadias are categorized into glanular, penile, and penopubic.^3^ Characteristics of male epispadias include a short phallus, abnormally located dorsal urethral meatus, dorsal chordee, and a ventrally hooded prepuce. Complete epispadias may involve external genitalia deformities, pubic symphysis diastasis, and urinary continence mechanism deficiency.^1^^,^^2^ Urinary incontinence is common in cases of penopubic or subsymphyseal epispadias, primarily due to bladder neck and striated sphincter deficiencies.^4^ In cases of penopubic or subsymphyseal epispadias, the urethra is entirely open, and the bladder outlet may be large enough that a finger can be inserted, indicating obvious incontinence.^4^

The primary goal of epispadias surgery is to restore the anatomical and functional aspects of the penis, with a focus on cosmetic appearance, urinary function, and sexual function.^1^^,^^5^ Bladder neck reconstruction is a surgical procedure that can restore continence function in cases of incontinence.^5^ This case report presents a unique instance of epispadias associated with ambiguous genitalia, where the corpora cavernosum and glans penis were concealed behind scrotal tissue, a phenomenon not previously reported in the literature.

## 2 Case Presentation

**Fig. 1:**
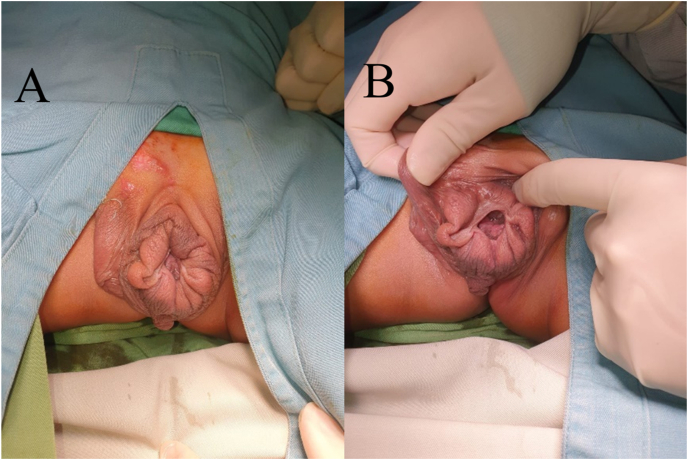
(A) Preoperative morphology of epispadia. (b) The wide opening of the external meatus.

**Fig. 2:**
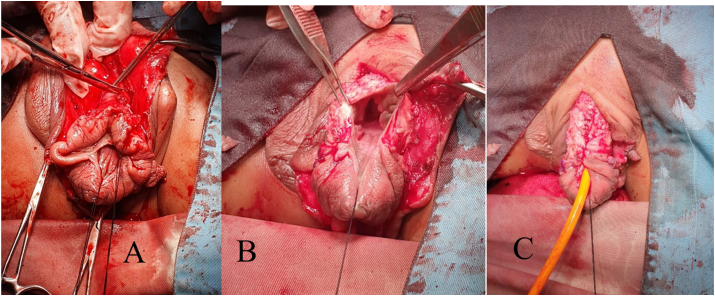
(A) After degloving penis, corpora of shaft penile was identified. (b) Glans were identified hidden by scroral skin. (c) Tubularization of the urethral plate, spongioplasty, corporoplasty with medial rotation of corporeal bodies, and glanuloplasty with meatoplasty.

**Fig. 3:**
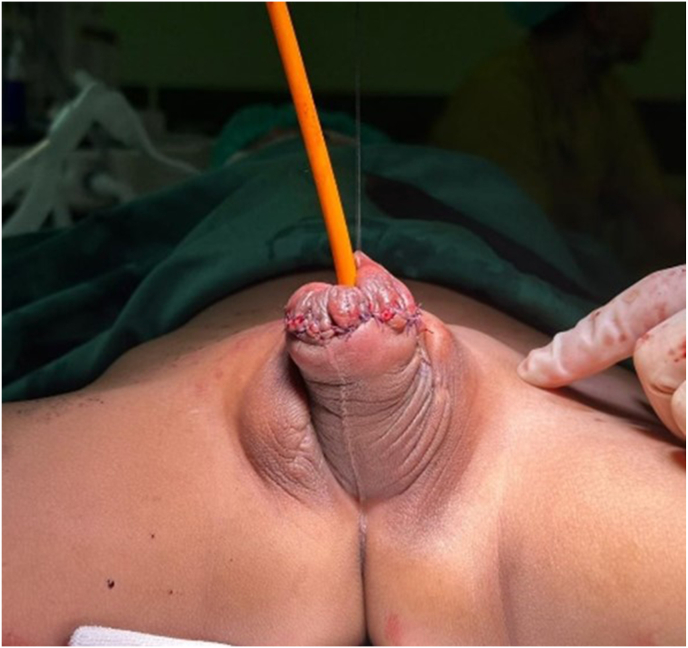
Post operative.

A 5-year-old male presented with an abnormal penile structure since birth, characterized by a dorsal penopubic urethral meatus. The penis resembled external female genitalia, with scrotal skin covering it (Fig. 1). Palpable testes were noted in the inguinal region. The wide-open meatus in the penopubic area led to urinary incontinence from birth. The patient underwent pelvic MRI which revealed the absence of a penis and diastasis of the pubic symphysis. Karyotyping examination confirmed a chromosomal pattern of 46XY. The patient underwent a two-stage operation, starting with bilateral orchidopexy to relocate both normal-sized testes to the scrotum. The second stage involved urethrocystoscopy, scrotoplasty, glanuloplasty, and penile reconstruction. The objective of the second stage procedure is to reconstruct the penis to restore its form and function. During the cystoscopy, it was discovered that the bladder neck was not fully intact, and there was a wide opening of the external urethral meatus due to which the vesical impression seemed empty and could not be filled. After the procedure, the scrotal skin was explored and the tissue was degloved. Upon release, structures resembling the corpus cavernosum were found, which widened laterally and separated. The corpus spongiosum and urethra were identified, but the dorsal part was absent until the proximal region (Fig. 2). After maximal degloving, the glans penis structure was discovered, with chordee present on the dorsal part. The degree of the chordee is less than 30°. Chordectomy was performed to form the body of the penis, and reconstruction was carried out from the glans penis and tubularization was performed to form the urethra and corpus spongiosum. Excess scrotal skin tissue is excised, and scrotoplasty is performed to create a cover of penile skin. It result the post-operative scrotum resembles a scrotal transposition. Both corpus cavernosum tissues were identified and appeared viable. A surgical procedure was performed to bring the urethral opening ventrally. This involved tubularization of the urethral plate, spongioplasty, corporoplasty with medial rotation of corporeal bodies, and glanuloplasty with meatoplasty (Fig. 2). The skin cover was achieved through the rotation of the ventral flaps and the use of z-plasty as needed. Tissue approximation from the neourethra and skin was done with PGA 5-0 sutures, with a z-plasty shape in the proximal part. The external urethral meatus was successfully moved to the distal with tubularization size of urethral catheter no 16Fr (Fig. 3). The surgical wound was closed with a dressing and covered with elastomul. On post-operative day (POD) 5, the dressing was removed, revealing a well-healed and dry surgical wound with no signs of infection.

The patient had follow-up appointments at the outpatient clinic on POD 14 and POD 30 to check the surgical wound. The wound was found to be intact and in good condition. The shape of the penis was normal, and the MUE was located at the glans penis. No leakage or fistula was found from the urethra. The patient and their family are satisfied with the appearance of the external genitalia post-operation. The next follow-up appointments will involve assessing urinary incontinence and bladder neck reconstruction.

## 3 Discussion

Epispadias is an uncommon congenital abnormality that is even rarer in isolated male cases.^6^ This condition falls under the BEEC spectrum, and the position of the meatus in epispadias can vary from glanular to penopubic.^6^^,^^7^ Penopubic-type epispadias cases always lead to urinary incontinence due to the absence of sphincter formation in the dorsal (roof) area. While incontinence has also been found in distal types, it is not as common as in penopubic types.^3^^,^^8^

Although many theories have been proposed to explain its occurrence, the exact cause of epispadias remains unclear.^2^ It is believed that abnormalities in the cloacal membrane may be responsible for its development. An abnormally large cloacal membrane can prevent the normal migration of mesenchymal tissue and is also prone to early rupture, which can lead to this spectrum of anomalies.^9^^,^^10^ Isolated epispadias occurs when this rupture causes a non-union of the distal portion of the urinary tract.^10^ Several animal models have also suggested that interactions between growth factors and transcription factors might contribute to its occurrence.^10^

The diagnosis of epispadias in males can be made based on physical examination. Common features of epispadias include a short and stubby phallus with a dorsal meatus, dorsal chordee, and ventrally hooded prepuce.^6^ Splaying and shortening of the corpora are also typically found in males with epispadias.^3^^,^^6^ Splaying occurs due to pubic diastasis, and the total corporal length is shorter due to the foreshortened anterior corporal segment, while the posterior segment is normal.^6^^,^^11^ The shorter appearance of the phallus is also due to the short urethral plate and dorsal chordee. All of these factors must be addressed during epispadias repair to ensure penile lengthening.^,^^10^^,^^11^ It's important to note that while the vas and ejaculatory ducts are normal in children with epispadias, they are at risk of injury during the reconstructive procedure.^7^

Typically, in cases of shaft penis epispadias, the scrotum appears normal and fully formed. However, in our unique case, the scrotum did not form normally, resulting in the structure of the shaft penis and glans being concealed behind the scrotal skin.

There are two surgical techniques used for epispadias, namely, the Cantwell-Ransley technique and the complete penile disassembly (Mitchell) technique.^5^^,^^10^ Both techniques have proven to be successful in restoring the anatomical shape and function of external genitalia.^3^^,^^8^ The ideal approach for male epispadias repair should aim to return the genitourinary anatomy to its normal location and configuration.^8^ This involves several goals such as correcting dorsal chordee, creating a straight urethra to allow easy catheterization or cystoscopy, achieving satisfactory cosmesis, minimizing complications, especially urethrocutaneous fistulas, maintaining erectile function, and creating urinary continence (penopubic epispadias).^7^ In our case, the penile structure was not visible because it was hidden behind the scrotal skin. After degloving, we found the structure of the corpus spongiosum and the penis with chordee. The surgical principle we followed was similar to the Cantwell-Ransley technique, where we tubularized the urethra after reconstructing both corpus cavernosum and glans penis until distal. We restored the urethra to its normal shape by performing tubularization with a 16Fr catheter size.

Epispadias surgery is typically performed within the first year of life and can be done in one or two stages.^1^ The primary goal of the first stage is to restore the anatomical shape of the penis and urethra, followed by a second stage which involves bladder neck reconstruction to improve continence.^1^ A single-stage procedure may also be selected, in which both bladder neck reconstruction and epispadias repair are performed simultaneously to correct incontinence as early as possible and reduce the likelihood of multiple surgeries.^1^^,^^9^

Complications may arise after undergoing epispadias repair. Some of these complications include the development of urethrocutaneous fistulas, persistent chordee, difficulty with urethral catheterization, and erectile dysfunction.^9^ Fistulas typically occur dorsally at the base of the penis where the tissue coverage is most delicate, and the corporal bodies do not yet cover the reconstructed urethra.^9^ The Cantwell–Ransley repair method has a fistula rate of 5%–20%, while the total penile disassembly technique has fistula rates of 10%–20%.^9^^,^^12^ A modified Cantwell–Ransley repair may require surgical reconstruction in 45% of patients, indicating a high rate of complications that require further surgical intervention.^9^ These complications are more common in patients who undergo the procedure as part of a staged exstrophy closure versus isolated epispadias.^9^ Even in experienced hands, epispadias repair is technically challenging, which contributes to the high rate of re-operation.^1^^,^^9^

Epispadias surgery has various long-term outcomes, including good cosmetic appearance, satisfaction with external genitalia after puberty, urinary continence ability, and sexual function of external genitalia. However, there is not enough data to determine satisfaction rates with cosmetic appearance. Continence success rates vary widely, ranging from as low as 50% to up to 90%.^13^^,^^14^ Research by Thomas et al. discovered that more than 80% of patients required re-operation to improve urinary continence through endoscopic agent injection or bladder neck reconstruction surgery.^9^ His research also found that 95% of patients could achieve a full erection, 26% experienced persistent dorsal chordee, and 43% experienced retrograde ejaculation.^9^^,^^11^ The numbers for continence success rates are well-known.^14^ Epispadias surgery is a challenging procedure, even for experienced urologists. Ideally, these surgeries should be performed at urology centers with pediatric urologists who have previously performed epispadias surgery.

## 4 Conclusion

Epispadias remains a challenging surgical endeavor, best performed by experienced pediatric urologists in specialized centers. Rare occurrences, such as our case with ambiguous genitalia, highlight the need for continued research to expand our understanding and refine surgical approaches. This study contributes to the existing literature and emphasizes the importance of a comprehensive approach to epispadias repair.

## Financial disclosure

None.

## CRediT authorship contribution statement

**Kevin Anthony Glorius Tampubolon:** Writing – review & editing, Writing – original draft, Methodology, Investigation, Data curation, Conceptualization. **Jupiter Sibarani:** Supervision, Methodology, Conceptualization.

## Declaration of competing interest

No conflict of interest.
